# Mouse models of telomere dysfunction phenocopy skeletal changes found in human age-related osteoporosis

**DOI:** 10.1242/dmm.014928

**Published:** 2014-03-13

**Authors:** Tracy A. Brennan, Kevin P. Egan, Carter M. Lindborg, Qijun Chen, Mariya T. Sweetwyne, Kurt D. Hankenson, Sharon X. Xie, Frederick B. Johnson, Robert J. Pignolo

**Affiliations:** 1 Department of Medicine, Perelman School of Medicine, University of Pennsylvania, Philadelphia, PA 19104, USA.; 2 Department of Pathology and Laboratory Medicine, University of Pennsylvania, Philadelphia, PA 19104, USA.; 3 Department of Clinical Studies-New Bolton Center and Animal Biology, School of Veterinary Medicine, University of Pennsylvania, Philadelphia, PA, USA.; 4 Department of Biostatistics and Epidemiology, Perelman School Of Medicine, University of Pennsylvania, Philadelphia, PA 19104, USA.; 5 Department of Orthopaedic Surgery, Perelman School of Medicine, University of Pennsylvania, Philadelphia, PA 19104, USA.

**Keywords:** Aging, Bone histomorphometry, Osteoporosis

## Abstract

A major medical challenge in the elderly is osteoporosis and the high risk of fracture. Telomere dysfunction is a cause of cellular senescence and telomere shortening, which occurs with age in cells from most human tissues, including bone. Telomere defects contribute to the pathogenesis of two progeroid disorders characterized by premature osteoporosis, Werner syndrome and dyskeratosis congenital. It is hypothesized that telomere shortening contributes to bone aging. We evaluated the skeletal phenotypes of mice with disrupted telomere maintenance mechanisms as models for human bone aging, including mutants in Werner helicase (*Wrn^−/−^*), telomerase (*Terc^−/−^*) and *Wrn^−/−^Terc^−/−^* double mutants. Compared with young wild-type (WT) mice, micro-computerized tomography analysis revealed that young *Terc^−/−^* and *Wrn^−/−^Terc^−/−^* mice have decreased trabecular bone volume, trabecular number and trabecular thickness, as well as increased trabecular spacing. In cortical bone, young *Terc^−/−^* and *Wrn^−/−^Terc^−/−^* mice have increased cortical thinning, and increased porosity relative to age-matched WT mice. These trabecular and cortical changes were accelerated with age in *Terc^−/−^* and *Wrn^−/−^Terc^−/−^* mice compared with older WT mice. Histological quantification of osteoblasts in aged mice showed a similar number of osteoblasts in all genotypes; however, significant decreases in osteoid, mineralization surface, mineral apposition rate and bone formation rate in older *Terc^−/−^* and *Wrn^−/−^Terc^−/−^* bone suggest that osteoblast dysfunction is a prominent feature of precocious aging in these mice. Except in the *Wrn^−/−^* single mutant, osteoclast number did not increase in any genotype. Significant alterations in mechanical parameters (structure model index, degree of anistrophy and moment of inertia) of the *Terc^−/−^* and *Wrn^−/−^Terc^−/−^* femurs compared with WT mice were also observed. Young *Wrn^−/−^Terc^−/−^* mice had a statistically significant increase in bone-marrow fat content compared with young WT mice, which remained elevated in aged double mutants. Taken together, our results suggest that *Terc^−/−^* and *Wrn^−/−^Terc^−/−^* mutants recapitulate the human bone aging phenotype and are useful models for studying age-related osteoporosis.

## INTRODUCTION

Human senile osteoporosis is generally characterized by low bone mass and altered microarchitectural features, primarily attributed to osteoblast dysfunction, which results in a substantially increased risk of fracture and subsequent disability (Kassem and Marie, 2011; Marie and Kassem, 2011a; Marie and Kassem, 2011b). This differs from well-studied postmenopausal osteoporosis, in which significant bone loss is attributed to increased osteoclast resorption concomitant with the loss of estrogen in women (Aaron et al., 1987; Rehman et al., 1994; Russo et al., 2003; Mullender et al., 2005; Pernow et al., 2009). One of the limitations to more intensive investigations of senile osteoporosis is the lack of physiologically relevant models of aging bone. Although the mechanisms of senile osteoporosis remain to be elucidated, some mechanistic clues have come from genetic conditions that potentiate telomere dysfunction. Two conditions of particular interest include dyskeratosis congenita (DC; incidence ~1 in 1 million) and Werner syndrome (WS; incidence 1 in 20,000–200,000), both of which are characterized by a complex phenotype including premature osteoporosis. In DC, osteoporosis is an accelerated form of that seen with physiological aging, whereas the osteoporosis of WS affects the limbs as well as the axial skeleton (Hofer et al., 2005; Mason et al., 2005). One of the most common forms of DC is caused by a mutation in *TERC* (the RNA template component of telomerase), which results in reduced telomerase activity. The lack of telomerase activity leads to prematurely shortened telomeres, telomere uncapping and the association of telomeric ends with DNA-damage proteins. Taken together, these findings suggest that dysfunctional telomeres likely are a major contributing factor to the pathology of DC, including osteoporosis (Mason et al., 2005).

WS is most commonly due to a loss-of-function mutation in *WRN*, which encodes a DNA helicase of the RecQ family and functions in the recombinational repair of stalled replication forks or double-strand breaks (Ozgenc and Loeb, 2005). Additionally, *WRN* is thought to play a role in telomere maintenance (Wyllie et al., 2000; Johnson et al., 2001; Opresko et al., 2002; Crabbe et al., 2004), which might be particularly important in situations when telomeres become dysfunctional, such as cellular aging.

In mice lacking telomerase and with shortened telomeres, *Wrn^−/−^* mutation results in an acceleration of defects seen in *Terc^−/−^* mutants and also the appearance of some pathologies typical of WS (e.g. osteoporosis) (Chang et al., 2004; Du et al., 2004). Previously, we and others found that deficiency in telomerase, alone or in combination with deficiency in the Werner helicase, leads to an accelerated low-bone-mass phenotype (Chang et al., 2004; Du et al., 2004; Pignolo et al., 2008; Saeed et al., 2011). Declines in mesenchymal progenitor cell number and in osteoblast differentiation are the major cellular mechanisms associated with premature bone loss (Pignolo et al., 2008; Wang et al., 2012). *In vitro*, osteoclast differentiation and function in *Wrn^−/−^*, *Terc^−/−^* and *Wrn^−/−^Terc^−/−^* mutants are comparable to those in age-matched wild-type (WT) mice (Pignolo et al., 2008; Wang et al., 2012).

**TRANSLATIONAL IMPACT**

**Clinical issue**

Human age-related osteoporosis poses a major problem to both elderly men and women. The low bone mass and altered microarchitectural features of the condition, which are mainly attributed to osteoblast dysfunction, increase the risk of bone fracture. It is predicted that the annual number of hip fractures worldwide due to osteoporosis will rise threefold from the 1990 figure of 1.7 million to 6.3 million by 2050. Therefore, it is crucial to develop novel therapies to help treat this condition. Although postmenopausal osteoporosis, which is attributed to increased osteoclast activity (osteoclasts destroy bone whereas osteoblasts build bone; normal bone is constantly remodeled by these two cell types), has been well studied, far less is known about the mechanisms of age-related osteoporosis. This is in part due to a lack of physiologically relevant models of this type of osteoporosis.

**Results**

Here, the authors investigate the premature osteoporosis that occurs in mouse models based on two human genetic progeroid (premature aging) conditions, Werner syndrome and dyskeratosis congenita. Telomere defects contribute to both of these disorders, and telomere shortening occurs with age in cells from most human tissues, including bone. It is possible, therefore, that telomere shortening contributes to bone aging. To test this hypothesis and to evaluate the physiological similarity of osteoporosis in *Terc^−/−^* mice (telomerase mutants; a model for dyskeratosis congenita), *Wrn^−/−^* mice (a model for Werner’s syndrome), *Terc^−/−^Wrn^−/−^* double-mutant mice and the human condition, the authors evaluate the skeletal phenotypes of the mutant mice. They show that these models reflect the major features of senile bone loss, including decreased trabecular and cortical bone volume, increased cortical porosity, and impairment of osteoblast function as a prominent cause of accelerated bone aging.

**Implications and future directions**

These findings suggest that *Terc^−/−^* and *Wrn^−/−^Terc^−/−^* mice recapitulate the bone changes that occur in aging human bones and establish these mice as appropriate models for studying senile osteoporosis. Moreover, they support the hypothesis that telomere-based mechanisms are important in bone aging. The models described will be useful for the identification and dissection of signaling pathways that are altered in response to telomere-based bone aging. Moreover, further studies incorporating these models should aid in the design and preclinical evaluation of potential therapeutic treatments for age-related bone loss.

Here, we test the hypothesis that deficiencies in *Terc^−/−^* and/or *Wrn^−/−^Terc^−/−^* mutant mice reflect many of the physiological features of human age-related osteoporosis, including decreased bone volume, osteoblast number and osteoblast function, as well as increased porosity and marrow adiposity. We present evidence that supports the use of *Terc^−/−^* and *Wrn^−/−^Terc^−/−^* mice as relevant models for studying human age-related osteoporosis and suggests that telomere-based mechanisms are important in the aging of bone tissue.

## RESULTS

### Telomere-based aging models show accelerated trabecular and cortical bone loss

**Fig. 1:**
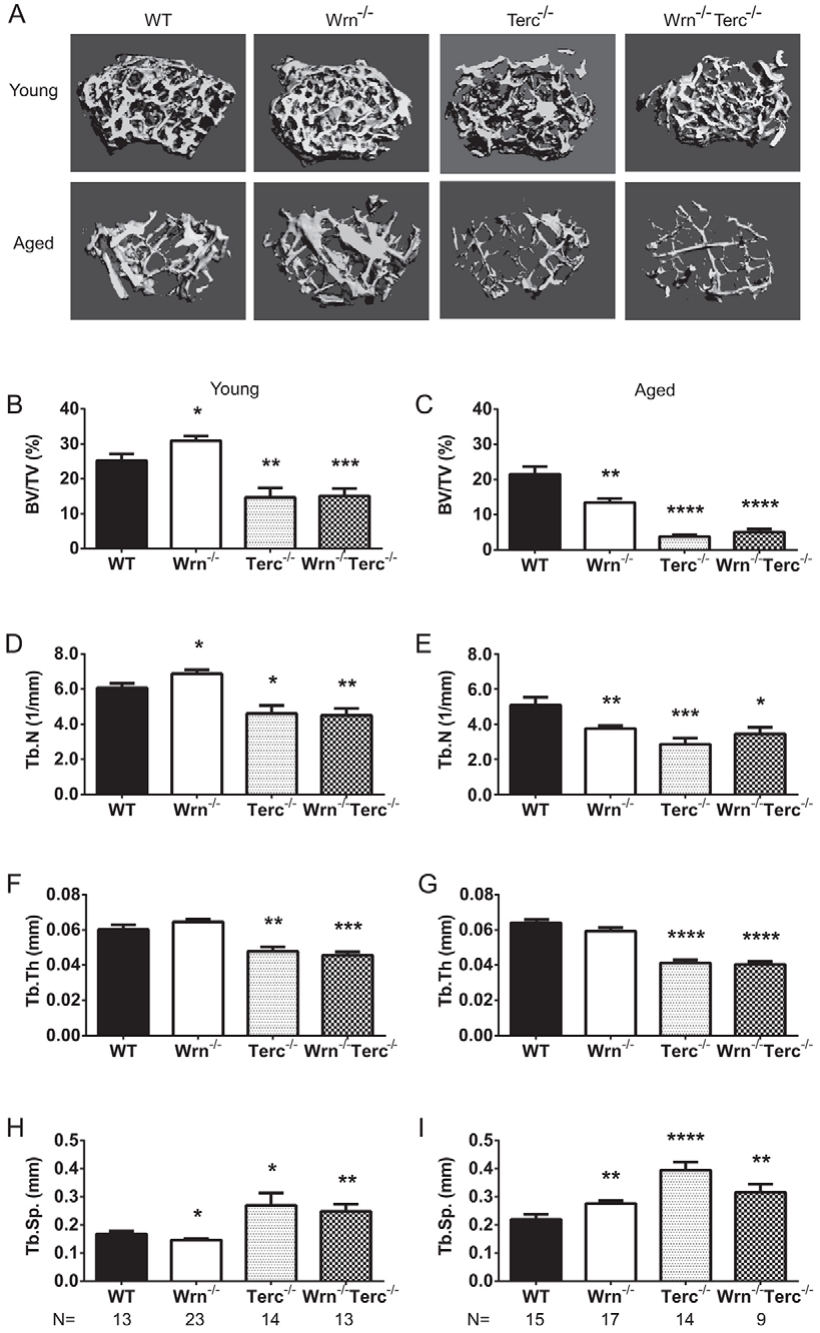
**Telomere-based changes in *Terc^−/−^* and *Wrn^−/−^Terc^−/−^* trabecular bone parameters**. (A) Three-dimensional representation of the trabecular ROI in the distal femurs. Distal femurs from young and aged male mice were evaluated for microarchitectural changes including (B,C) bone volume/total volume (BV/TV; %), (D,E) trabecular number (Tb. N; /mm), (F,G) trabecular thickness (Tb. Th; mm) and (H,I) trabecular separation (Tb. Sp.; mm). Data represent means ± s.e.m. Statistical significance is *P<0.05, **P<0.005, ***P<0.0001 and ****P<0.0001 compared with WT in each age group.

A hallmark of human senile osteoporosis is decreased bone volume, primarily attributed to decreased trabecular bone, which can also be accompanied by decreased cortical bone volume (Parfitt, 1984; Kleerekoper et al., 1985; Rehman et al., 1994; Mullender et al., 2005; Pernow et al., 2009). High-resolution micro-computerized tomography (μCT) was used for analysis of the distal femur from young and aged WT, *Wrn^−/−^*, *Terc^−/−^* and *Wrn^−/−^Terc^−/−^* mice. Fig. 1A shows representative images of the trabecular region of interest (ROI) for each of the genotypes in the young and aged groups. Bone volume declined with age across the genotypes (age, *P*<0.0001; genotype, *P*<0.0001; interaction, *P*=0.0012; Fig. 1B,C). At 3 months of age, *Terc^−/−^* and *Wrn^−/−^Terc^−/−^* femurs had statistically significant decreases in bone volume (41.7% and 47.6%, respectively) compared with the age-matched WT (Fig. 1B). This comparative deficit became more pronounced with age, as reflected in Fig. 1C. In the aged group, *Wrn^−/−^*, *Terc^−/−^* and *Wrn^−/−^Terc^−/−^* mice had statistically significant decreases in bone volume (46.4%, 84.9% and 69.8%, respectively) compared with aged WT. The decreases in bone volume in young (Fig. 1B) and aged (Fig. 1C) *Terc^−/−^* and *Wrn^−/−^Terc^−/−^* were accompanied by significantly decreased trabecular number (age, *P*<0.0001; genotype, *P*<0.0001; interaction, *P*=0.0027; Fig. 1D,E) and trabecular thickness (age, *P*=0.0248; genotype, *P*<0.0001; interaction, *P*=0.0498; Fig. 1F,G), with a commensurate increase in trabecular separation {age, *P*<0.0001; genotype, *P*<0.0001; interaction, *P*=0.1941 [nonsignificant (ns)]; Fig. 1H,I}.

Moreover, when the bone volume of young *Wrn^−/−^Terc^−/−^* was compared with WT aged mice, *Wrn^−/−^Terc^−/−^* (*P*=0.01) bone volume was significantly decreased. This accelerated bone loss in young *Wrn^−/−^Terc^−/−^* was accompanied by trends toward both decreased trabecular number and trabecular separation as well as a significant decrease in trabecular thickness (*P*<0.0001).

**Fig. 2:**
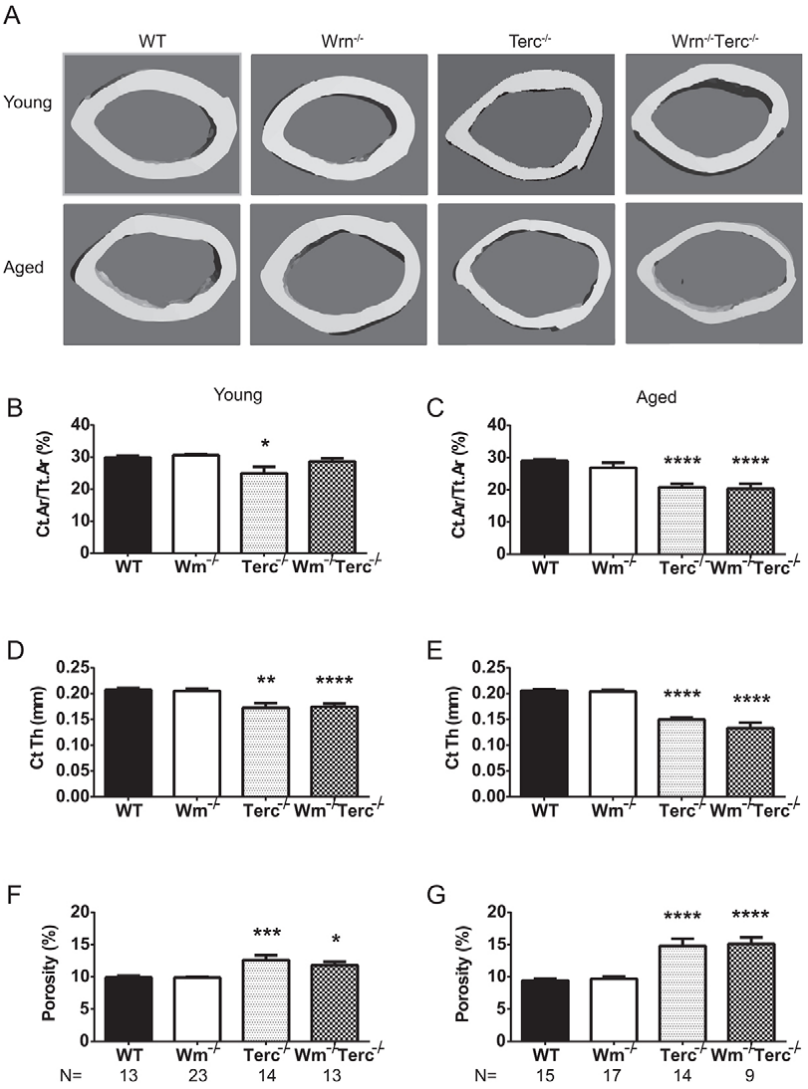
**Telomere-based changes in *Terc^−/−^* and *Wrn^−/−^Terc^−/−^* cortical parameters.** (A) Three-dimensional representation of the cortical ROI in the femur. The femoral midshaft from young and aged male mice were evaluated for cortical bone changes including (B,C) cortical area/total area (Ct. Ar/Tt. Ar; %), (D,E) cortical thickness (Ct. Th; mm) and (F,G) total pore volume/bone volume (porosity; %). Data represent means ± s.e.m. Statistical significance is *P<0.05, **P<0.005, ***P<0.0025, ****P<0.0001, compared with WT in each age group.

Human senile osteoporosis can be accompanied by thinning of the cortical shell and increased cortical porosity causing an increased risk of fracture (Feik et al., 1997; Bousson et al., 2001; Ostertag et al., 2009; Nishiyama et al., 2010; Zebaze et al., 2010). Cortical bone was analyzed for both young and aged mice at the femoral midshaft using μCT. Fig. 2A shows representative images of the cortical ROI. The cortical bone area decreased significantly with age and genotype [age, *P*<0.0001; genotype, *P*<0.001; interaction, *P*=0.067 (ns)]. Unlike the trabecular changes, in young mice only *Terc^−/−^* showed a decrease (4.0%) in cortical area compared with age-matched WT (Fig. 2A,B). However, significant decreases in cortical area were seen in aged *Terc^−/−^* and *Wrn^−/−^Terc^−/−^* mice compared with older WT mice (28.2% and 29.7%, respectively; Fig. 2C). In addition to changes in cortical area, cortical thickness significantly decreased (age, *P*=0.0003; genotype, *P*<0.0001; interaction, *P*=0.0037). The cortical thickness of *Terc^−/−^* and *Wrn^−/−^Terc^−/−^* mutants was significantly decreased in both young and aged groups compared with their respective WT counterparts (Fig. 2D,E). The effects of accelerated aging in the young *Terc^−/−^* and *Wrn^−/−^Terc^−/−^* femurs were further highlighted by their statistically significant decrease in cortical thickness compared with aged WT femurs (*P*=0.0021 and *P*=0.0001, respectively). Although the cortical thickness decreased with age for *Terc^−/−^* and *Wrn^−/−^Terc^−/−^* femurs, there were no statistically significant differences in the endosteal [age, *P*=0.0012; genotype, *P*=0.7552 (ns); interaction, *P*=0.6157 (ns)] or periosteal [age, *P*=0.0302; genotype, *P*=0.1594 (ns); interaction, *P*=0.2842 (ns)] surfaces between these genotypes compared with young or aged WT. Average animal weight within young (28.5±1.4 g, WT; 28.2±0.6 g, *Wrn^−/−^*; 25.6±1.3 g, *Terc^−/−^*; 24.4±1.4 g, *Wrn^−/−^Terc^−/−^*) and older (32.8±0.8 g, WT; 32.2±0.7 g, *Wrn^−/−^*; 29.0±2.0 g, *Terc^−/−^*; 30.2±2.0 g, *Wrn^−/−^Terc^−/−^*) groups were not statistically significant compared with WT.

Cortical porosity increased with age and genotype (age, *P*=0.0038; genotype, *P*<0.0001; interaction, *P*=0.0022). Relative to the WT mice, porosity increased in *Terc^−/−^* and *Wrn^−/−^Terc^−/−^* mutants, in both young (27.1% and 19.0%, respectively) and aged (49.3% and 60.1%, respectively) groups (Fig. 2F,G). When compared with aged WT femurs, porosity in the cortical bone of either young *Terc^−/−^* or young *Wrn^−/−^Terc^−/−^* mice was significantly increased (*P*=0.0008 and *P*=0.0024, respectively).

Taken together, our data show that the accelerated bone aging in *Terc^−/−^* and *Wrn^−/−^Terc^−/−^* mouse models not only displays characteristic features of human senile osteoporosis, as examined at the structural level by μCT, but that these characteristics present earlier and in general are more pronounced than in older WT mice.

### Osteoblast dysfunction is the primary cellular mechanism for osteoporosis in telomere-based bone aging

**Fig. 3:**
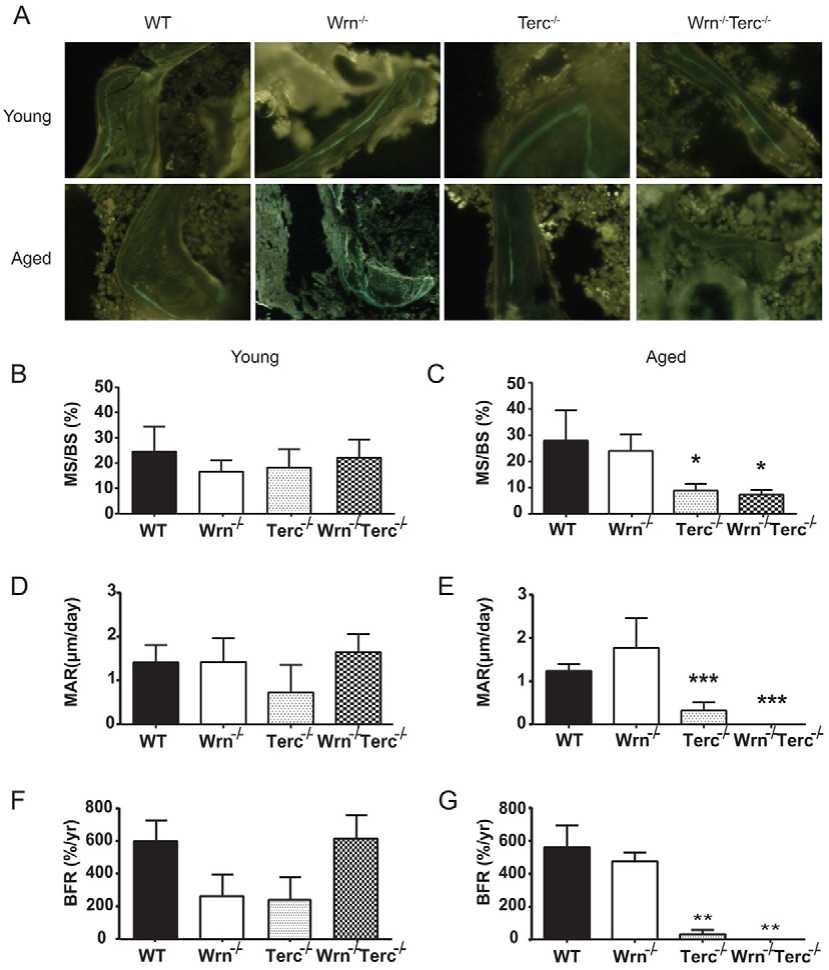
**Decreased kinetic parameters of bone formation in aged *Terc****^−/−^* **and *Wrn****^−/−^****Terc****^−/−^* **mice.** (A) Representative fluorescently labeled sections of metaphyseal distal femurs for young and aged WT, *Wrn^−/−^*, *Terc^−/−^* and *Wrn^−/−^Terc^−/−^* animals. (B,C) Mineralizing surface/bone surface (MS/BS; %), (D,E) mineral apposition rate (MAR; μm/day) and (F,G) bone formation rate (BFR; %/yr) are shown. Data represent means ± s.e.m. Statistical significance is *P<0.05, **P<0.01 and ***P<0.0025, compared with WT in each age group (*n*=5 per group).

The decrease in bone volume seen in human senile osteoporosis is generally attributed to decreased bone formation due to osteoblast dysfunction, in contrast to menopausal osteoporosis in which increased osteoclast resorption seems to be the predominant mechanism (Rehman et al., 1994; Clarke et al., 1996; Pernow et al., 2009). Therefore, to assess osteoblast function, calcein double labeling was used to quantify dynamic parameters of bone formation in young and aged mice (Fig. 3A). Although the differences observed in mineralized surface [mineralized surface (MS)/bone surface (BS), %] [age, *P*=0.0861 (ns); genotype, *P*=0.0495; interaction, *P*=0.0091; Fig. 3B,C] and bone formation rate (BFR; %/year) [age, *P*=0.1213 (ns); genotype, *P*=0.0061; interaction, *P*=0.0137; Fig. 3F,G] were related to telomere-based defects alone, decreases in mineral apposition rate (MAR; μm/day) were influenced by both age and genotype (age, *P*=0.0074; genotype, *P*=0.0004; interaction, *P*=0.0031; Fig. 3D,E). Young *Wrn^−/−^*, *Terc^−/−^* and *Wrn^−/−^Terc^−/−^* mice showed no statistically significant differences in MS, MAR or BFR compared with young WT mice (Fig. 3B,D,F). However, MS, MAR and BFR significantly and dramatically declined in aged *Terc^−/−^* and *Wrn^−/−^Terc^−/−^* mice compared with WT mice that were on average more than 30% older (Fig. 3C,E,G).

**Fig. 4:**
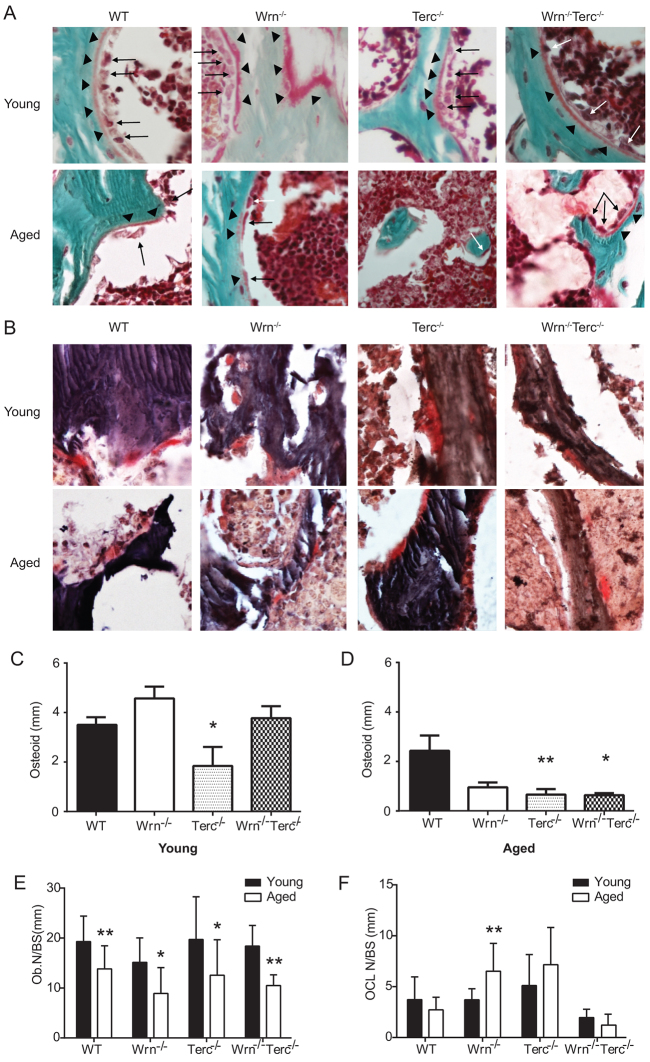
**Declines in osteoblast number and osteoid, but not osteoclast number, in aging bone.** (A,B) Representative sections of metaphyseal distal femurs from young and aged WT, *Wrn^−/−^*, *Terc^−/−^* and *Wrn^−/−^Terc^−/−^* mice stained with Goldner’s trichrome (A; arrowheads indicate osteoid; arrows indicate osteoblasts) and tartrate-resistant acid phosphatase (TRAP; B). (C,D) Decreased osteoid is present in young *Terc^−/−^* and older *Terc^−/−^* and *Wrn^−/−^Terc^−/−^* mice compared with WT. Data represent means ± s.e.m. Statistical significance is *P<0.05 and **P<0.01, compared with WT in each age group (*n*=5 per group). (E) Decreased osteoblast number per mm bone surface (Ob. N/BS) occurs in wild-type and mutant genotypes with age. (F) Unchanged number of osteoclasts per mm bone surface (OCL N/BS) is seen with aging in WT, *Terc^−/−^* and *Wrn^−/−^Terc^−/−^* mice (*n*≥5 samples). (E,F) Statistical significance is *P<0.05 and **P<0.01, compared with young genotype-matched group (*n*=5 per group).

Concomitant with decreased kinetic parameters of bone formation, osteoid significantly decreased in the aged *Terc^−/−^* and *Wrn^−/−^Terc^−/−^* femurs compared with WT (age, *P*<0.0001; genotype, *P*=0.0008; interaction, *P*=0.0350; Fig. 4A,C,D). At the cellular level, osteoblast numbers decreased significantly with age and across all genotypes [age, *P*<0.0001; genotype, *P*=0.0700 (ns); interaction, *P*=0.9236 (ns); Fig. 4E]. Importantly, only the *Wrn^−/−^* single mutant had a statistically significant increase in osteoclast number with age [age, *P*=0.1764 (ns); genotype, *P*<0.0001; interaction, *P*=0.0150; Fig. 4B,F]. Thus, taken together with their profoundly decreased capacity for osteoid production, mineralization and bone formation, this strongly suggests that, like in human senile bone loss, osteoblast dysfunction is the primary mechanism for osteoporosis in *Terc^−/−^* and *Wrn^−/−^Terc^−/−^* mutants.

**Fig. 5:**
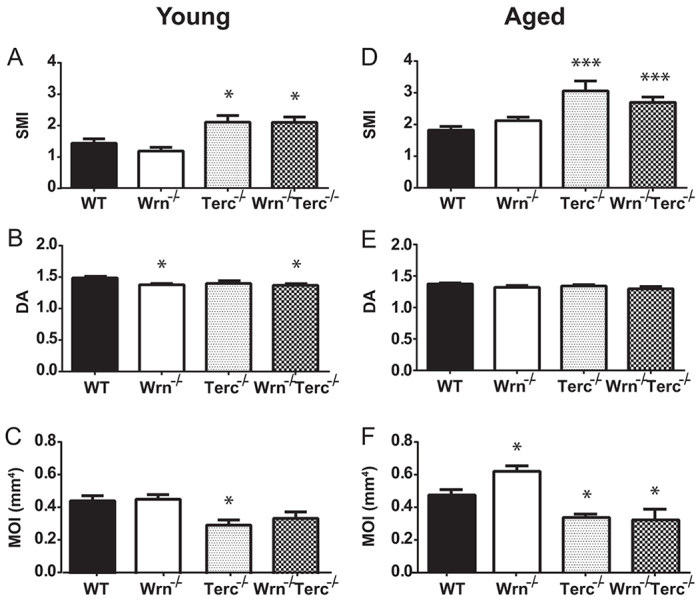
**Mechanical alterations in *Terc^−/−^* and *Wrn^−/−^Terc^−/−^* bone accompany their osteoporotic phenotype.** Trabecular bone analysis of young and aged femurs for (A,D) structure model index (SMI) and (B,E) degree of anisotropy (DA). (C,F) Cortical moment of inertia (MOI; mm^4^) is decreased in young *Terc^−/−^* and older *Terc^−/−^* and *Wrn^−/−^Terc^−/−^* mice compared with WT. Data represent means ± s.e.m. Statistical significance is *P<0.05 and ***P<0.0001 compared with WT in each age group.

Osteoblast dysfunction in the *Terc^−/−^* and *Wrn^−/−^Terc^−/−^* mutants was accompanied by alterations in mechanical properties. Young *Terc^−/−^* and *Wrn^−/−^Terc^−/−^* mutant femurs had a statistically significant increase in structure model index (SMI), which continued to increase with age [age, *P*<0.0001; genotype, *P*<0.0001; interaction, *P*=0.2378 (ns); Fig. 5A,D] and indicating a more rodlike trabecular structure. The degree of anisotropy (DA) was statistically decreased in young *Wrn^−/−^* and *Wrn^−/−^Terc^−/−^* mutants [age, *P*=0.0004; genotype, *P*=0.0078; interaction, *P*=0.7226 (ns); Fig. 5B,E]. The moment of inertia (MOI) was statistically decreased in young *Terc^−/−^* femurs and, in the aged group, the MOI significantly decreased in both *Terc^−/−^* and *Wrn^−/−^Terc^−/−^* mutants [interaction, *P*=0.1002 (ns); genotype, *P*<0.0001; age, *P*=0.1898 (ns), Fig. 5C,F]. These changes in mechanical parameters are consistent with those seen in aging humans (Bono and Einhorn, 2003; Russo et al., 2003; Jiang et al., 2005).

### High bone-marrow adiposity in *Terc^−/−^* and *Wrn^−/−^Terc^−/−^* mutants

**Fig. 6:**
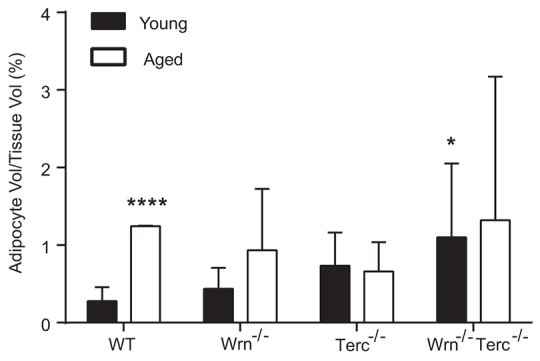
**Increase in bone-marrow fat content in *Wrn****^−/−^****Terc****^−/−^* **mutants.** Adipocyte volume was measured in WT, *Wrn^−/−^*, *Terc^−/−^* and *Wrn^−/−^Terc^−/−^* femurs from young and aged animals. Data represent average ± s.e.m. Statistical significance is ****P<0.0001 or *P<0.05 compared with young WT (*n*=5 per group).

Adiposity increases with human bone aging and more severely with osteoporosis (Justesen et al., 2001; Rosen and Bouxsein, 2006). Our analysis of adiposity showed no relationship with aging and/or genotype on percent adipose tissue [age, *P*=0.1027 (ns); genotype, *P*=0.3390 (ns); interaction, *P*=0.5074 (ns)]. However, an analysis of adipocyte volume in young *Wrn^−/−^Terc^−/−^* mice compared with young WT animals demonstrated that the former have a statistically significant increase in bone-marrow fat content (*P*<0.05), which remained elevated in aged double mutants (Fig. 6). In addition, aged *Terc^−/−^* and *Wrn^−/−^Terc^−/−^* mice had bone-marrow fat content similar to much older WT mice.

## DISCUSSION

Senile osteoporosis results in a substantially increased risk of fracture and subsequent disability (Raisz and Rodan, 2003). Although postmenopausal osteoporosis has been well studied, far less is known about the mechanisms of age-related osteoporosis affecting both men and women. In this study, we assessed telomere-based aging mouse models according to key characteristics of human senile bone loss, both to evaluate their physiological similarities to the human condition as well as to substantiate the importance of telomere dysfunction as a mechanism for skeletal aging. Although previous data suggest that telomerase-based models of accelerated aging have osteoporosis, our current study shows that these models reflect the major features of senile bone loss, and that impairment of osteoblast function is a prominent aspect of accelerated bone aging. Inadequate osteoblast-mediated mineralization during bone remodeling is likely to be responsible for osteopenia in mouse models of physiologic as well as premature aging (Jilka, 2013).

Bone loss in both trabecular and cortical locations is a hallmark of age-related osteoporosis. Similar to human senile osteoporosis, *Terc^−/−^* and *Wrn^−/−^Terc^−/−^* mice had decreased trabecular bone volume and trabecular number (Table 1). A significant decrease in *Terc^−/−^* trabecular bone has also been reported (Saeed et al., 2011) from histological analysis in the spinal region (L5 vertebra). Although trabecular thickness also decreased with age in our models, no consensus has been reached for humans, where reports have shown a decline in trabecular thickness (Parfitt et al., 1983; Birkenhäger-Frenkel et al., 1988), a decline in men only (Wakamatsu and Sissons, 1969; Aaron et al., 1987; Mellish et al., 1989), and a decline in both men and women (Rehman et al., 1994). These discrepancies could be attributed to human variation; however, when looking at severely osteoporotic male bone samples, Pernow et al. showed that trabecular thickness was significantly decreased (Pernow et al., 2009).

**Table 1:**
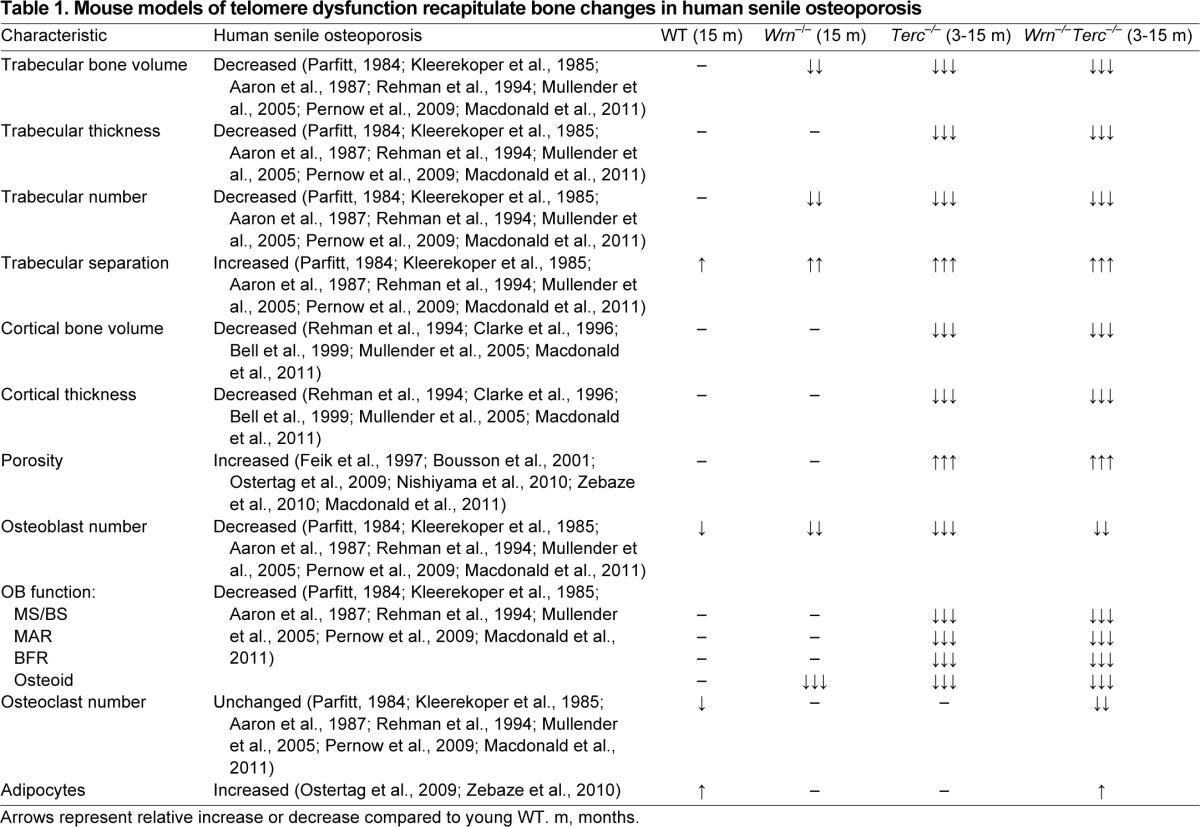
Mouse models of telomere dysfunction recapitulate bone changes in human senile osteoporosis

Cortical bone loss in human senile osteoporosis is structurally identified by cortical thinning and increased porosity (Feik et al., 1997; Ostertag et al., 2009; Nishiyama et al., 2010; Zebaze et al., 2010; Macdonald et al., 2011). μCT analysis of *Terc^−/−^* and *Wrn^−/−^Terc^−/−^* mice showed that they recapitulate this phenotype by having both a significant decrease in cortical thickness and a significant increase in porosity, which together contributed to the significant loss of cortical bone volume compared with WT mice (Table 1).

Age-related bone loss in humans is attributed to osteoblast dysfunction in the presence of normal osteoclast activity (Marie and Kassem, 2011b). Although the number of osteoblasts present in the aged mice was comparable across genotypes, parameters of bone formation (including osteoid, mineralization, MAR and BFR) were significantly decreased in both *Terc^−/−^* and *Wrn^−/−^Terc^−/−^* mice compared with the WT counterparts (Table 1). Taken together, our data suggest that osteoblasts present in the *Terc^−/−^* and *Wrn^−/−^Terc^−/−^* mice are dysfunctional. This is supported by similar data reported for the analysis of tibiae in *Terc^−/−^* mice (Saeed et al., 2011). In addition, our previous work using *Terc^−/−^* and *Wrn^−/−^Terc^−/−^* mice demonstrated dysfunctional osteoblast differentiation *in vitro*, independent of cell proliferation (Pignolo et al., 2008; Wang et al., 2012). The contribution of osteoblast dysfunction to these osteoporotic phenotypes is further highlighted by the fact that TRAP^+^ osteoclasts in *Terc^−/−^* and *Wrn^−/−^Terc^−/−^* bones remained relatively unchanged in the young versus older groups. This is supported by our previous work that demonstrated that osteoclast activity was similar in WT, *Terc^−/−^* and *Wrn^−/−^Terc^−/−^* osteoclasts *in vitro* (Wang et al., 2012). Thus, telomere-based osteoporosis is primarily due to age-related osteoblast dysfunction in the context of unchanged osteoclast function.

Osteoblast dysfunction coincides with deleterious changes in mechanical properties of bone. The *Terc^−/−^* and *Wrn^−/−^Terc^−/−^* trabecular bone had statistically increased SMIs in both young and aged groups, consistent with changes seen in aging bone that demonstrate a transition from a more plate-like trabecular structure to a more rod-like structure typical of osteoporotic bone (Ding and Hvid, 2000; Chen et al., 2013). Although the DA varies with skeletal site, with human bone aging there is initially an increase in anisotropy followed by a period of trabecular perforation and an eventual decrease in anisotropy (Jiang et al., 2005). In young *Wrn^−/−^* and *Wrn^−/−^Terc^−/−^* mutants, the DA is decreased and this level is maintained with aging in these genotypes. Furthermore, the MOI was significantly decreased in *Terc^−/−^* and *Wrn^−/−^Terc^−/−^* cortical bone. This decrease in MOI is characteristic of osteoporotic bone that is more easily fractured (Bono and Einhorn, 2003; Russo et al., 2003).

In bone, there seems to be a reciprocal relationship between the number of osteoblasts and adipocytes, with increased bone-marrow adiposity manifesting as another prominent feature of human senile osteoporosis (Justesen et al., 2001; Rosen and Bouxsein, 2006). Aged WT bone marrow had a significant increase in adiposity. By 3 months of age, however, *Wrn^−/−^Terc^−/−^* mice already had a significantly increased volume of bone-marrow adipose tissue, and young *Terc^−/−^* mice displayed a trend toward increased bone-marrow adiposity compared with WT counterparts.

Age-related osteoporosis is a complex condition that requires physiologically relevant models. An early model of senile bone loss involved using mouse strains that had a low peak bone mass, including C57BL/6J, that would develop osteoporosis naturally with age (Matsushita et al., 1986; Perkins et al., 1994; Bergman et al., 1996; Takeda et al., 1997b; Takeda et al., 1997a; Takeda et al., 1997c; Cao et al., 2003; Ferguson et al., 2003). However, the long time over which these strains develop characteristic features of bone aging make these models less appealing. To obviate this problem, strategies were implemented to mimic bone aging through ovariectomy, orchiectomy or bone-marrow ablation (Wink and Felts, 1980; Kalu, 1984; Liang et al., 1992; Mosekilde et al., 1994; Zhang et al., 1998). Although these approaches will cause an osteoporotic phenotype, they are better models for secondary causes of bone loss (e.g. sex-hormone deficiency) rather than primary age-related osteoporosis. Advantages and limitations of mouse models for the study of bone aging, and their applicability to the human condition, have been comprehensively reviewed (Jilka, 2013).

The use of genetically modified animals has enabled the development of osteoporotic mouse models (Kawaguchi et al., 1999; Vogel et al., 1999; Li et al., 2000; Bonyadi et al., 2003; Ito et al., 2003; Sun et al., 2004; Rasheed et al., 2006), but raises the question of how to qualify genetic changes as relevant to age-related bone loss specifically. The gene(s) modified in a proposed model of senile osteoporosis should (a) effect osteoblast differentiation and/or function; (b) accelerate the processes naturally impacted by aging, preferably on both the cellular and organismal level; and (c) result in a bone phenotype that recapitulates aspects of age-related bone loss in humans. Previous research has validated the influence of the *Wrn* and *Terc* genes on aging (Epstein et al., 1966; Chang et al., 2004; Mason et al., 2005; Ozgenc and Loeb, 2005). The results of the present study demonstrate that the *Terc^−/−^* and *Wrn^−/−^Terc^−/−^* mutations in mice cause a bone phenotype very similar to that seen in age-related bone loss in humans (Table 1), including osteoblast dysfunction, and thus are appropriate models for studying senile osteoporosis.

## MATERIALS AND METHODS

### Animals

The University of Pennsylvania Institutional Animal Care and Use Committee approved the use of mice described in this study. Mutant mice had the *Wrn^−/−^* and *Terc^−/−^* alleles backcrossed on the C57BL/6J background (for >11 generations). Fourth-generation (G4) *Wrn^−/−^Terc^−/−^* mice were produced as previously described (Pignolo et al., 2008; Wang et al., 2012). WT mice were obtained via standard matings using the C57Bl/6J strain that was used for backcrossing the *Wrn^−/−^* and *Terc^−/−^* alleles. *Wrn^−/−^* mutants were generated by crossing *Wrn^+/−^* mutants. *Terc^−/−^* and *Wrn^−/−^Terc^−/−^* mice used in experiments were from G4 lineages. Young animals across all genotypes were used in experiments at 3 months of age. Aged WT and *Wrn^−/−^* mutant animals were sacrificed at 15 months. *Terc^−/−^* and *Wrn^−/−^Terc^−/−^* mutants were sacrificed by 15 months or earlier, when they exhibited signs of significant suffering and impending demise including severe lethargy and/or major (>15%) weight loss. Based on our previous experience, animals exhibiting these moribund features died within 1 week. The average age of the older *Terc^−/−^* group was 11 months of age and that of the older *Wrn^−/−^Terc^−/−^* group 10 months of age.

### μCT analysis

#### Trabecular and cortical bone

High-resolution images of femurs were acquired by using a Scanco VivaCT 40 device (Bruettisellen, Switzerland). The femurs were scanned with a source voltage of 55 kV, a source current of 142 μA and an isotropic voxel size of 10.5 μm. After scanning, three-dimensional image data was reconstructed and structural indices were calculated using Scanco μCT V6.1 software.

The area for trabecular analysis started 57.5 μm proximal from the growth plate at the distal end of the femur and extended proximally 1050 μm toward the femoral head. The ROI for measurement of trabecular microarchitectural variables was defined manually by outlining the bone within the endocortical margins and allowing a few voxels between the ROI and the endocortical margin, as described previously (Bouxsein et al., 2010). An upper threshold of 1000 Hounsfield units and a lower threshold of 220 Hounsfield units were used to delineate each pixel as ‘bone’ or ‘non-bone’. Representative three-dimensional cross-sectional images for each genotype within the young and aged groups are shown in gray scale. Trabecular bone volume per total volume (BV/TV), mean trabecular thickness (Tb. Th), mean trabecular number (Tb. N, and mean trabecular separation (Tb. Sp) were calculated using Scanco μCT V6.1 software.

The cortical ROI was defined as extending 262.5 μm distally and 262.5 μm proximally from the midshaft. An upper threshold of 1000 Hounsfield units and a lower threshold of 260 Hounsfield units were used to delineate each pixel as ‘bone’ or ‘non-bone’. Cortical area per total area (Ct. Ar/Tt. Ar), average cortical thickness (Ct. Th), periosteal surface and endosteal surface were calculated using Scanco μCT V6.1 software.

#### Porosity

High-resolution images of femurs were acquired by using a Scanco μCT 35 device (Bruettisellen, Switzerland). The femurs were scanned with a source voltage of 55 kV, a source current of 142 μA and an isotropic voxel size of 6 μm. Porosity was assessed from the midshaft and extending 600 μm toward the proximal femur head. An upper threshold of 1000 Hounsfield units and a lower threshold of 447 Hounsfield units were used to delineate each pixel as ‘bone’ or ‘pores’. After scanning, porosity was calculated using Scanco μCT V6.1 software.

### Histological analysis

To measure dynamic bone formation parameters, mice were injected intraperitoneally with calcein (Sigma, St Louis, MO, USA; 30 mg/kg body weight) and xylenol orange (Sigma, St Louis, MO, USA; 90 mg/kg body weight) on day 9 and day 2 before tissue harvest.

Mouse hind limbs were excised, cleaned of soft tissue and fixed in 3.7% formaldehyde for 72 hours. Isolated bone tissue was dehydrated in graded alcohols (70 to 100%), cleared in xylene and embedded in methyl methacrylate (80% methyl methacrylate, 20% dibutyl phthalate, 2.5% benzoyl peroxide) by standard methods. Femurs placed in plastic blocks were cut longitudinally to expose trabecular bone using a Polycut-S motorized microtome (Reichert-Jung, Nossloch, Germany). Consecutive 5-μm sections were collected beginning 25 μm into trabecular bone for histological analysis. For all animals, except those in the aged *Terc^−/−^* and *Wrn^−/−^Terc^−/−^* groups, at least nine consecutive sections were used for analysis; in the latter two groups, at least 18 consecutive sections were used.

Femur sections were deplasticized in xylene and assessed for kinetic parameters of bone formation. Selected ROIs (100 μm distal to the growth plate and 50 μm in from the endosteal cortical bone) were visualized using a Nikon Eclipse 90i microscope. Image capture was performed using NIS Elements Imaging Software 3.10 Sp2 and a Nikon DS-Fi1 camera using Nikon 4×/0.2 Plan Apo and 40×/0.95 Plan Apo objectives. The Bioquant Osteo II digitizing system (R&M Biometrics, Nashville, TN, USA) was used for image analysis according to the manufacturer’s instructions. Mineralizing surface (MS/BS %) and MAR (μm/day) were quantified from ROIs in trabecular bone using a Nikon 20×/0.75 Plan Apo objective. BFR was calculated from mineralizing surface and MAR. Femur tissue sections were then stained with Goldner’s Trichrome or TRAP as previously described (Brennan et al., 2011; Singh et al., 2013). Osteoid (mm), osteoblast number (Ob N/BS, /mm) and osteoclast number (OCL N/BS,/mm) were quantified within the ROI described above (Parfitt et al., 1987) using Nikon 40×/0.95 Plan Apo objectives. Adipocytes (adipocyte vol/tissue vol) were identified morphologically and quantified as previously described (Elbaz et al., 2009) using a Nikon 20×/0.75 Plan Apo objective. Image capture was performed as described above.

### Statistics

To determine the effects of genotypes, two-way analysis of variance (ANOVA) was used with the following factors: age (young and aged) and genotype (WT, *Wrn^−/−^*, *Terc^−/−^*, *Wrn^−/−^Terc^−/−^*). The Tukey post-hoc method for multiple comparisons was used to compare results among young WT, *Wrn^−/−^*, *Terc^−/−^* and *Wrn^−/−^Terc^−/−^* mice, as well as among aged WT, *Wrn^−/−^*, *Terc^−/−^* and *Wrn^−/−^Terc^−/−^* mice (within group comparison), and to compare between age-grouped genotypes (intergroup comparison). All statistical analyses were performed using GraphPad Prism 4.0 software (San Diego, CA, USA). An adjusted *P*-value of <0.05 was considered significant for all analyses. All statistical tests are two-sided. Data are represented as mean ± standard error of the mean (s.e.m.).
